# A Treatise on Diseases of the Skin, with Special Reference to Their Diagnosis and Treatment

**Published:** 1887-06

**Authors:** 


A TREATISE ON DISEASES OF THE SKIN, WITH SPECIAL REFERENCE TO THEIR DIAGNOSIS AND TREATMENT. *By* T. M'CALL ANDERSON, M.D., *with colored plates and other illustrations. Pp. xviii and 662, Philadelphia:* P. BLAKISTON, SON & CO., *Chicago:* W. T. KEENER, *1887.*

This volume is a welcome addition to the not over large list of treatises on its subject. For more than a quarter of a century the author has had exceptional opportunities for the study of skin diseases, and he has an added advantage, as an author, in the fact that he is also a general practitioner and holds the chair of clinical medicine in the university of Glasgow.

Many of the articles upon diseases not endemic in England, or upon others, as ulcers, upon which the author is not as well qualified to write, are written by able men whose residence abroad, or whose special experience in practice specially fits them for the work. The pathology and treatments given are thoroughly in accord with the most recent and accepted opinions. The arrangement of topics, the indices, the printing, paper and binding are all excellent. The book may fairly be said to be the most satisfactory English treatise upon the subject. E. W.

